# The subsurface lesion in erosive tooth wear

**DOI:** 10.1016/j.jdent.2023.104652

**Published:** 2023-09

**Authors:** S.P. Jadeja, A. LeBlanc, S. O'Toole, R.S. Austin, D. Bartlett

**Affiliations:** Faculty of Dentistry, Oral and Clinical Sciences, Centre for Clinical, Oral and Translational Sciences, King's College London, London SE1 9RT, United Kingdom

**Keywords:** Enamel, Profilometry, Abrasion, Erosion, SEM

## Abstract

**Objectives:**

This study compared the surface change on natural and polished enamel exposed to a joint mechanical and chemical wear regimen.

**Methods:**

Human enamel samples were randomly assigned to natural (*n* = 30) or polished (*n* = 30) groups, subjected to erosion (*n* = 10, 0.3% citric acid, 5 min), abrasion (*n* = 10, 30 s), or a combination (*n* = 10). Wear in the form of step height was measured with a non-contact profilometer, and surface changes were inspected with SEM on selected sections. Data was normalised and underwent repeated measures MANOVA, accounting for substrate and erosive challenge as independent variables, with Bonferroni correction for significant post hoc interactions.

**Results:**

After four cycles, polished samples had mean step heights of 3.08 (0.40) μm after erosion and 4.08 (0.37) μm after erosion/abrasion. For natural samples, these measurements were 1.52 (0.22) μm and 3.62 (0.39) μm, respectively. Natural surfaces displayed less wear than polished surfaces under erosion-only conditions (*p*<0.0001), but the difference disappeared with added abrasion. SEM revealed a shallow subsurface layer for polished surfaces and natural ones undergoing only erosion. However, natural surfaces exposed to both erosion and abrasion showed deeper subsurface changes up to 50 µm.

**Conclusion:**

Natural enamel, when exposed to erosion alone, showed less wear and minimal subsurface alterations. But with added abrasion, natural enamel surfaces saw increased wear and notable subsurface changes compared to polished ones.

**Clinical Significance:**

The pronounced subsurface lesions observed on eroded/abraded natural enamel surfaces highlight how combined wear challenges may accelerate tooth tissue loss.

## 1 Introduction

Erosive tooth wear is thought to be a surface phenomenon with limited sub-surface destruction. Surprisingly, there is limited indication of the depth of the impact of acid challenges on the subsurface depth of enamel, with research tending to focus on quantitative surface methodologies [1]. Although microhardness gives an indication of destruction below the surface, it is widely recognised to have a plateauing effect and the relationship between wear and softening is not linear [2]. This non-linear relationship has been attributed to individual factors, such as the chemistry of saliva and the nature and preparation of the tooth tissue overall along with the specific model perameters including the storage of samples [3]. Furthermore, most erosion studies have utilised flattened, polished enamel surfaces to allow for consistent measurements [4], [5], [6] but un-altered, natural enamel is thought to react differently to erosive tooth wear [7]. When considering erosion specifically, one study observed the solubility and rate of dissolution of human tooth enamel increased with deeper layers of enamel were tested [8]. Furthermore, Wong et al. demonstrated how the outermost layer of enamel, referred to as the aprismatic layer, played a significant role in the resistance against dental caries due to its strong resistance against acid dissolution [9]. One of the hypothesised reasons for this is the aprismatic region's higher mineral content in the outer 10–30 µm of the enamel [10,11]. In addition, the polishing process inevitably removes this layer, alters the enamel surface structure, and introduces cracks that potentially modify the behaviour of the surface and subsurface reaction to an erosive attack [12]. Evidence from our group has suggested that in early surface wear (5 min of acid exposure) there is prismatic destruction rather than immediate opening of prisms [13]. Natural enamel surfaces have been shown to wear at a slower rate than in polished enamel. However, the subsurface differences between polished and natural enamel have yet to be identified in detail.

Erosive challenges on enamel have been demonstrated to affect the subsurface layer through demineralisation, however most research has focused on polished surfaces, which would have exposed prisms to acid challenge [14], [15], [16], [17], [18]. In these studies, the authors have used both qualitative (scanning electron microscopy (SEM) imaging and microhardness [15]) and quantitative measurements (optical coherence tomography (OCT) [18]) to assess the subsurface effects. Eisenburger et al. performed SEM imaging on a cross section of eroded polished enamel with and without ultra-sonification [14]. They observed that prism destruction was visible 9–12 µm below the surface before ultra-sonification and 3–4 µm after ultra-sonification. This implies that there is a subsurface layer of affected of enamel, but also that it is susceptible to mechanical disturbances. Lussi et al. [16] also performed cross-sectional SEM imaging on polished eroded enamel who observed heavily demineralised prisms, followed by a lesser affected area before sound enamel was reached [16]. This layer also appeared to extend 9–12 µm below the surface [16].

The only study investigating the subsurface layer on natural surfaces to the authors knowledge is an *in-vivo* study by Austin et al. who used Swept Source-OCT to examine early erosion on natural enamel surfaces [19]. They observed a heightened OCT signal intensity following exposure to orange juice, suggesting acid-induced changes in sub-surface enamel to about 33 µm depth. However, the study could not provide detailed sub-surface structure or the extent of the erosive damage, suggesting a need for further in-depth studies or additional imaging techniques.

Our ability to measure surface wear on natural enamel with profilometry has improved. Digital profiles can now be registered, and mathematical techniques used precisely analyses differences between sequential scans [20]. Subtracting scan data facilitates the calculation of bulk wear by enabling a step height calculation using the ISO 5436–1 standard [20,21], with an ability to detect 1 μm enamel loss on natural enamel after exposure to 0.3% critic acid [22,23].

Therefore, the aim of this present study, was to compare differences between natural and polished enamel after combined mechanical abrasion and chemical erosion. The first null hypothesis proposed that there would be no difference in observed wear in the form of surface step height between natural and polished surfaces when exposed to erosion and erosion/abrasion challenges. The second null hypothesis was that there would be no difference in the depth of the subsurface layer on natural surfaces and polished surfaces.

## 2 Methods

The primary measurement outcome between groups was wear in the form of step height, which is widely recognised as the gold standard in quantifying bulk enamel loss [22,23]. Data from a previous study comparing natural and polished surfaces using profilometry [13] in GPower 3.1.9.6 yielded a high effect size of 3.6. Using a repeated measures MANOVA at 90% power with 6 different test groups indicated that an overall sample size of 12 was needed. As previous data was limited to erosion only, we increased this to 60 samples to ensure we were powered to determine the effect between abrasion and erosion on natural surfaces.

**Fig. 1:**
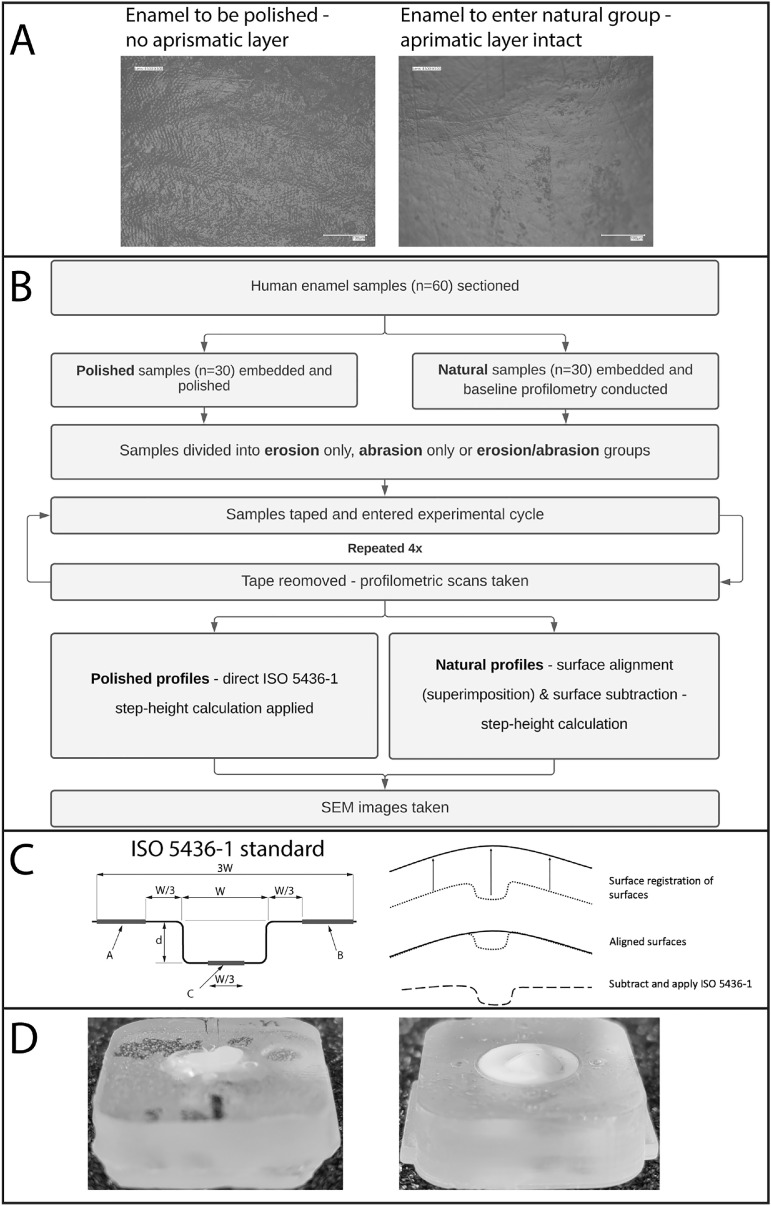
Outlines the study's workflow. (A) presents 500x co-axial microscopy images of enamel samples before preparation, ensuring that natural samples started the study with an aprismatic layer of enamel. (B) is a flowchart summarising the methodological sequence. (C) illustrates the ISO-5436–1 standard and a simplified diagram of its application to a natural surface, following the superimposition/subtraction workflow. (D) shows both polished and natural samples at a macro level to provide context for the curvature present in the natural surface.

The method workflow is summarised in Fig. 1. Non-carious, human enamel samples (*n* = 60) were acquired from previously extracted caries-free human molars under ethical approval and guidelines (REC: 12/LO/1836) and with patient consent. Samples were examined under a Keyence VHX-7000 digital microscope (Keyence UK, Milton Keynes, UK) at 500x magnification under co-axial light to ensure the surfaces were free of cracks and to determine samples which had maintained the aprismatic layer (Fig. 1A). Samples were decoronated along the cemento-enamel junction and sectioned along the mesio-distal and bucco–lingual planes using water-cooled wafer diamond blades (Labcut 1010, Agar Scientific Limited, UK). Following sectioning, buccal samples were assigned to polished (*n* = 30) or natural (unpolished) (*n* = 30) groups and then embedded into self-curing bis-acrylic models compatible with the DentaGen V.1.50 Syndicad brushing machine (Syndicad Ingenieurburo, Munchen, Germany) and each measured 5 × 25 × 20 mm. Polished samples (*n* = 30) underwent previously published protocols to remove 250–300 µm of the surface enamel and produced a flatness tolerance of ±0.4 μm [24]. Finally, the samples were ultrasonicated (GP-70, Nusonics, Lakewood, USA) in 100 ml deionised water for 15 min and air-dried for 24 h using previously published protocols [13,24]. The natural surfaces human enamel samples were mounted into a 3D-printed UV cured acrylic resin model compatible with a DentaGen V.1.50 Syndicad brushing machine.

Baseline measurements were taken using a 655 nm white light confocal profilometer (WLCP) mounted on an automatic motion system (XYRIS 2000CL, Taicaan, Southampton, UK) [25]. Samples were randomly assigned into subgroups of 10: erosion, abrasion, and erosion with abrasion for natural and polished enamel.

Reference zones were created on the polished and natural enamel samples using PVC tape (3 M, UK), the reference zones followed a previously used by Mylonas et al. investigating both polished and natural surfaces [13]. For polished samples two protected reference areas were created on either side of the enamel and exposed a 1 mm window for the tooth wear challenge to take effect, leaving a refence area to allow for the ISO 5436–1 step height parameters to be applied (Fig. 1C). This was to prevent artefacts formed during scanning such as the batwing effect influencing the measured step height when quantifying the bulk enamel wear [26]. For the natural enamel surfaces a 1.5 mm diameter hole was created in the tape and used to expose the zenith of the enamel surface towards the mid-buccal of the sample. Once taped samples were immersed for 5 mins in 100 ml of 0.3% at pH 2.7 citric acid [27,28] under constant agitation at 60 rpm (Stuart Scientific, Mini Orbital Shaker SO5, Bibby) [13]. Samples were then removed and immersed into deionised water for 60 s followed by a 1-hour water bath. The reference PVC tape was removed carefully, cleaned with ethanol to remove surface deposits, and left to air dry for 24 h to enable re-scanning under the WLCP [20]. This process was repeated for 4 cycles using fresh acid at each cycle. For the erosion abrasion group, after the erosion challenge, samples were subjected to an additional 30 strokes of toothbrush abrasion at 1 linear stroke per second using an automatic tooth-brushing machine (DentaGen V.1.50 Syndicad, Germany), with an Oral B P40 toothbrush (ADA reference toothbrush) at a load between 290 - 295 g with a toothpaste slurry made up of 3 parts artificial saliva to 1 parts fluoride free toothpaste [1]. Each sample was cycled four times using the same procedure scanning and retaping in between, with a replenished slurry each time.

Scans were imported into a software (Geomagic Inc, North Carolina, USA) as x, y, z point clouds, wrapped to create a mesh and a process of superimposition/surface registration was performed using an iterative closest point (ICP) for best fit alignment [20,22]. Following the initial alignment, irregular datapoints and artefacts were removed around the scan peripheries and a ICP alignment was repeated, a final alignment was performed with the exclusion of the wear scar area and only alignment to reference area was performed [29]*.* The transformed scan data pairs were imported into a metrology software (Mountains8, Digitalsurf, France) capable of performing surface subtraction. The workflow provided data which could be expressed as step height allowing for the use of the ISO 5436–1 calculation (summarised in Fig. 1B) to accurately quantify the induced wear post challenge.

The visual characteristics of both the natural and polished enamel exposed to erosion and erosion/abrasion was further explored using imaging via a scanning electron microscopy (SEM) [14,30]. This was taken to understand what qualitative changes may have occurred to the enamel prism below the surfaces in direct contact with the erosive challenges. Randomly selected polished and natural samples from both erosion and erosion/abrasion groups were embedded in epoxy resin (EpoThin2, Buehler, ITW Test & Measurement GmbH, Leinfelden-Echterdingen, Germany), placed under vacuum, followed by positive pressure to ensure good edge retention of the resin during sectioning. Samples were left for 24 h to harden and then sectioned perpendicularly through the lesion (in a buccolingual direction in the mesiodsital plane) using a Buehler IsoMet 1000 wafer blade sectioning saw (Buehler, ITW Test & Measurement GmbH, Leinfelden-Echterdingen, Germany). Finally, samples were ground using a Laboforce-100TM (Struers, Cleveland, USA) using 1200, 2000, 4000 grit silicone-carbide disks followed by polishing cloths to remove any scratches caused by the section process. One half of each section was then placed in 1 M HCL for 15 s to enhance the visualisation of the enamel prism morphology under SEM. Samples were mounted to SEM stubs using carbon tape and placed into a Leica EM ACE600 (Leica Microsystems UK Ltd, Milton Keynes, United Kingdom) sputter coater and coated with a 6 nm of gold to prevent electron charging of the tooth surface. They were then placed into a benchtop SEM (JCM-7000 NeoScope Benchtop SEM, JEOL LTD, Heartfordshire, UK) and imaged with 10–15 keV between 55 and 1300 x magnification. Imaging of the enamel surface both at the lesion location and the adjacent reference area of the enamel to distinguish between reference area enamel and the lesion.

Data from the scans were analysed in SPSS (IBM Corporation, Armonck, USA) and tested for normality using Shapiro-Wilks test and visually using histograms and boxplots and found not to be normal and were log transformed. A repeated measures MANOVA was performed using substrate (polished enamel and natural enamel) and wear challenge (erosion, abrasion, erosion/abrasion) as the independent variables for each time point. As significant differences between the substrate and challenge were observed (*p* < 0.001), further post hoc analysis with Bonferroni correction was performed between groups and timepoints.

## 3 Results

**Fig. 2:**
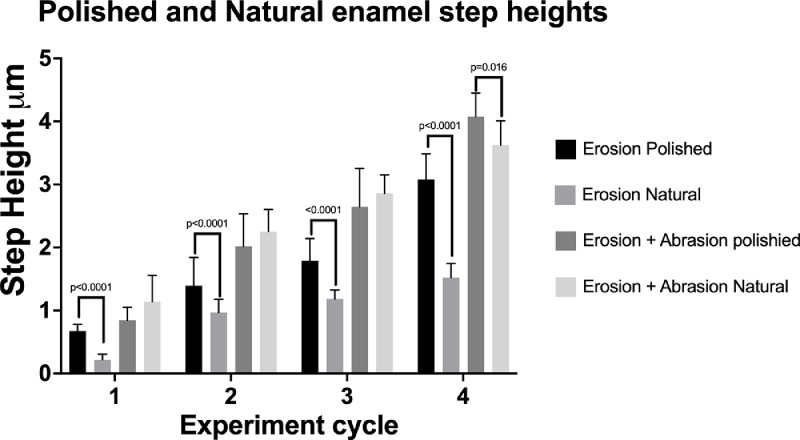
Comparison of polished vs natural enamel following progressive step height change for erosion only group and erosion + abrasion group.

The mean step height values are summarised in Table 1 representing the bulk enamel wear . There were statistically significant differences between natural and polished surfaces (*p* < 0.001) after erosion, with greater step heights recorded on the polished compared to the natural surfaces. For samples exposed to erosion/abrasion there was only a significant difference observed in the final cycle (*p* = 0.016) this has been visually represented in Fig. 2. The abrasion-only data from both polished and natural surfaces demonstrated shallow step heights due to potentially undetectable enamel loss, or positive step heights resulting from possible build-up of debris.

**Table 1:** Mean step height (and standard deviation) in µm, representing enamel wear, for all four experimental cycles on both polished and natural enamel samples. 'X' in the natural group, subjected to abrasion only, denotes instances where no measurement could be recorded.

	Erosion		Abrasion		Erosion/Abrasion	
Experiment cycle	Polished	Natural	Polished	Natural	Polished	Natural
1	0.66 (0.11)	0.22 (0.09)	0.05 (0.36)	X	0.85 (0.21)	1.14 (0.41)
2	1.39 (0.45)	0.97 (0.21)	0.17 (0.50)	X	2.02 (0.52)	2.25 (0.35)
3	1.79 (0.35)	1.18 (0.14)	0.19 (0.44)	X	2.65 (0.61)	2.86 (0.29)
4	3.08 (0.40)	1.52 (0.22)	0.26 (0.29)	0.14 (0.04)	4.08 (0.37)	3.62 (0.39)

**Fig. 3:**
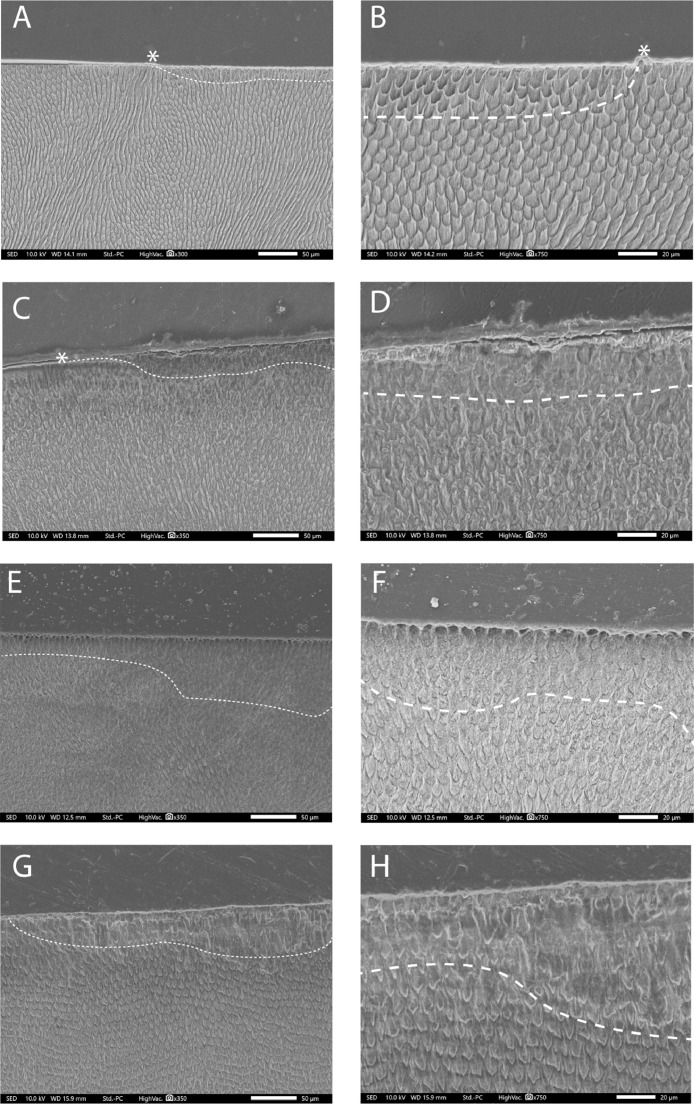
SEM comparisons of prism structure in each treatment group (all samples acid-etched with HCL to reveal prism morphology The figures on the left (A,C,E,G) show x350 magnification and the figures on the right (B,D,F,H) show x750 magnification of polished eroded enamel, natural eroded enamel, polished eroded abraded enamel and natural eroded abraded enamel respectively. The dashed line depicts where the depth to which the subsurface prisms have been effected.

SEM images were taken to provide qualitative images to investigate the underlying morphology of the prismatic and interprismatic enamel be observed for samples exposed to erosion and erosion/abrasion in Fig. 3. The Figures on the left (A,C,E,G) show x350 magnification and the figures on the right (B,D,F,H) show x750 magnification of polished eroded enamel, natural eroded enamel, polished eroded abraded enamel and natural eroded abraded enamel respectively, the dashed line represents the subsurface area that displayed changes to the prismatic structure.

Polished, eroded enamel showed evidence of underlying prism disruption at depths of approximately 10–20 µm below the surface, with the asterisk demarking where the boundary between the reference and lesion surfaces was visible (Fig. 3A and B). On natural, eroded enamel, there was a less pronounced change beyond the immediate impact on the surface of the enamel; however there was still a region of relatively poorly defined prisms directly below the eroded surface to around 30 µm in depth (Fig. 3C and D). This region was not present in other regions of the tooth beyond the lesion subsurface. For the polished, eroded and abraded enamel, it was difficult to discern clear changes on the subsurface of the enamel but there appeared to be a reduced contrast ranging between 10 and 50 µm of prisms beneath the lesion (Fig. 3E and F). However, the eroded and abraded natural enamel demonstrated a more apparent deformation of the prisms and deep subsurface change up to 50 µm (Fig. 3G and H).

## 4 Discussion

In contrast to previous studies, when erosion and abrasion challenges were performed, the step height data of the natural and polished surfaces were not statistically different until the final experimental cycle. We therefore partially rejected the first null hypothesis. The step height for erosion on natural surfaces was significantly lower than that of the polished surfaces throughout all experimental the cycles, which supports the existing literature [13,20]. Fig. 2 illustrates that by the final cycle, the enamel loss (as measured in step height) in the natural erosion group was approximately half of that in the polished erosion group. However, this proportional difference was not observed in the erosion/abrasion groups. This subsequently prompted further exploration of the groups that were exposed to erosion to investigate how the mechanical process of brushing is impacting the enamel structure below the challenged surface. When examining the sub surface lesion on the natural surface, the SEM images demonstrated minimal change for the erosion-only samples with the prism structure appearing to have slightly less definition of prism structures under the challenge site. However, significant sub surface change was observed on the erosion/abrasion challenge, with what appeared to be deeper deformation of the prisms structure. This complements previous findings where initial prism destruction occurs in early wear and gives an insight into why the results with erosion and abrasion were observed. A possible explanation is that the repeated mechanical conditioning via abrasion following the erosion challenge may have introduced spaces within and between prisms, facilitating deeper penetration of the acid. Prisms are already exposed on polished enamel, which explains their increased vulnerability to the erosive challenge. This study demonstrates that the outer layer of natural enamel retains structural integrity after erosion yet is still vulnerable to mechanical wear. This concept has been proposed and discussed before [6,7,17]; however this study demonstrates how erosion on polished surfaces misrepresents the change that may be occurring in the natural oral environment. Given that the polishing process removes around 250–300 µm of surface enamel the results conform to Theuns et al. [8] who observed that enamel dissolution increases the closer in proximity to the dentin. On a more positive note, this may prove promising for remineralisation clinically. It is this softened layer of enamel that fluoride-based products are targeting, yet, delivery is typically through mechanical brushing [31,32]. What was unexpected was the demonstration that the mechanical action appears to be a necessary element to expose prisms, facilitating subsurface softening. In this experiment it caused the outer surface of both polished and natural enamel to wear in a comparable manner. It appears that mechanical abrasion following chemical erosion removes the outer layer, making the surface equally susceptible to wear as seen on the polished enamel samples.

Ganss et al. compared erosion depths on human natural enamel and polished enamel after 3 h of citric acid exposure [7]. The wear lesions were at least 10 times larger than those in this paper, but they observed a difference between polished and natural surfaces with lesion depth of 115.0 (44.6) μm and 70.3 (16.2) μm respectively. To calculate the depth of the wear lesion, the authors used a regression line to estimate the pre-eroded height of the enamel and then erosion depth was determined as the vertical distance between the highest point on a single reference surface to the lowest point of the eroded surface. This method did not utilise data derived from a step height calculation but used mathematical interpolations to approximate for the differences between the highest point of the reference area and the lowest point of the wear lesion.

We also observed significant differences in measured wear between the natural and polished enamel groups for erosion. After four cycles of erosion, the lesions on polished enamel were almost twice as deep as those on natural enamel surfaces. Standard polishing techniques remove the natural outer surface of enamel, making the exposed prisms more susceptible to acids [6], which emphasises the importance of preserving this outer layer to assess its true clinical impact. The fact that removal of surface enamel increasing vulnerability to dissolution has been highlighted by other groups. Whereas, the erosion on the natural surface was comparatively small, the combination of erosion and abrasion negates the resistance of the surface and yielded values closer to those of polished surfaces. It seems likely that the mechanical action of brushing together with erosion of the mineral rich surface was sufficient to cause significantly more wear.

SEM imagery facilitated investigation into the natural subsurface of enamel. The authors hypothesised the possibility of a distinct area of deteriorated enamel prisms beneath the region subjected to erosion, however this layer was not as clearly evident in the natural erosion only group, with only a subtle change in the definition of the prims extending to depths similarly observed by Austin et al. [19]. However, a more apparent subsurface layer was observed on the natural erosion/abrasion images, where prism definition and arrangement appeared to have been lost to a depth of around 50 µm. This suggests that the natural enamel maintains structure following erosion, accounting for the shallower step height, while still vulnerable to mechanical force. This may explain why comparable step heights between both natural and polished erosion/abrasion groups were observed along with increasing the susceptibly of the underlying prisms to demineralisation. Further work is required with quantification of the mineral content in the progressing deeper layers of the enamel to confirm the exact potential for surface recovery following remineralisation.

The limitation of this study resides primarily in the fact that it was an *in vitro* study without the inclusion of natural saliva. Theoretically, this subsurface lesion could be confirmed with an *ex-vivo* model. The use of software for sequential surface registration, especially those employing an iterative closest point (ICP) algorithm, is not optimal due to its inherent limitations. Nevertheless, by employing a subtraction method, many of these initial scan registration inaccuracies can be mitigated, provided the registration process itself is conducted under supervision and not solely guided by the ICP algorithm [21]. The potential for bias was itself balanced by randomising the samples. Further shortcomings of this study model are that it is a highly simplified representation of the oral environment by using artificial saliva, a fluoride-free toothpaste slurry, and limiting abrasion to linear manual brush strokes. This may be influencing the response both of the step height recorded as well as the SEM images obtained.

Despite the limitations, this study enabled an investigation into the early erosion and abrasion subsurface lesions on natural enamel samples. The findings support the evidence that the erosive tooth wear process on enamel is driven by the chemical wear and exacerbated by mechanical wear. It also supports that natural surfaces have a greater resistance to erosion based wear than polished enamel yet both appeared susceptible to mechanical forces following acid exposure.

## 5 Conclusion and future work

This study demonstrates the significant role played by the outermost layer of enamel in resisting dietary based acids to tooth wear, opening the research space to further exploration of this layer, particularly in field of tooth wear. It also emphasises the potential impact mechanical wear may have on eroded enamel and its increasing susceptibility to future erosive wear challenges. This could have significant implications for how preventive advice and treatment modalities need to be delivered. Further elemental analysis will aid in qualifying to what extent the eroded enamel has demineralised and determine the impacts of possible remineralisation agents. Finally, this paper presented the application of a novel method to capture step height on natural surfaces and utilise it for chemo mechanical effects.

## CRediT authorship contribution statement

**S.P. Jadeja:** Conceptualization, Methodology, Data curation, Visualization, Investigation, Formal analysis, Writing – original draft. **A. LeBlanc:** Methodology, Data curation, Validation, Visualization, Investigation, Writing – review & editing, Formal analysis, Resources. **S. O'Toole:** Methodology, Formal analysis, Validation, Data curation, Writing – review & editing. **R.S. Austin:** Supervision, Methodology, Validation, Data curation. **D. Bartlett:** Conceptualization, Supervision, Writing – review & editing.

## Declaration of Competing Interest

The authors declare that they have no known competing interests or personal relationships that has influenced the work presented in this paper. The authors would like to acknowledge the BBSRC iCase (Grant No. BB/M009513/1) award with Unilever Oral Care (Grant No. MA-2019–0247 N) for their support.
